# Projecting the potential impact of the Cap-Score™ on clinical pregnancy, live births, and medical costs in couples with unexplained infertility

**DOI:** 10.1007/s10815-017-1021-4

**Published:** 2017-09-23

**Authors:** Joseph B. Babigumira, Fady I. Sharara, Louis P. Garrison

**Affiliations:** 1 Global Medicines Program, Department of Global Health, University of Washington, 1510 San Juan Road, Box 357965, Seattle, WA 98092 USA; 2 Pharmaceutical Outcomes Research and Policy Program, Department of Pharmacy, University of Washington, Seattle, WA USA; 3 Virginia Center for Reproductive Medicine, Reston, VA USA; 4 Department of Obstetrics and Gynecology, George Washington University, Washington, DC USA

**Keywords:** Sperm capacitation, Costs, Clinical pregnancy, Live births, Decision-analytic modeling

## Abstract

**Purpose:**

The Cap-Score™ was developed to assess the capacitation status of men, thereby enabling personalized management of unexplained infertility by choosing timed intrauterine insemination (IUI), versus immediate in vitro fertilization (IVF) or intracytoplasmic sperm injection (ICSI) in individuals with a low Cap-Score™. The objective of this study was to estimate the differences in outcomes and costs comparing the use of the Cap-Score™ with timed IUI (CS-TI) and the standard of care (SOC), which was assumed to be three IUI cycles followed by three IVF-ICSI cycles.

**Methods:**

We developed and parameterized a decision-analytic model of management of unexplained infertility for women based on data from the published literature. We calculated the clinical pregnancy rates, live birth rates, and medical costs comparing CS-TI and SOC. We used Monte Carlo simulation to quantify uncertainty in projected estimates and performed univariate sensitivity analysis.

**Results:**

Compared to SOC, CS-TI was projected to increase the pregnancy rate by 1–26%, marginally reduce live birth rates by 1–3% in couples with women below 40 years, increase live birth rates by 3–7% in couples with women over 40 years, reduce mean medical costs by $4000–$19,200, reduce IUI costs by $600–$1370, and reduce IVF costs by $3400–$17,800, depending on the woman’s age.

**Conclusion:**

The Cap-Score™ is a potentially valuable clinical tool for management of unexplained infertility because it is projected to improve clinical pregnancy rates, save money, and, depending on the price of the test, increase access to treatment for infertility.

## Introduction

The median global prevalence of infertility, defined as the inability to get pregnant after at least 12 consecutive months of unprotected sex, is approximately 9–15% [1, 2]. This corresponds to somewhere from 40 to 70 million couples [1, 2]. In the USA, approximately one million women are infertile by this definition [3]. However, a larger number of women, 7.5 million, have impaired fecundity—the reduced ability to get pregnant or carry a baby to term [3]. Among men, some 9.4% are sub-fertile or non-surgically sterile [4].

Of the couples who are unable to conceive, a male factor is present in 20–70% [5]. As many as 50% of the cases of male factor infertility are undetectable using traditional semen analysis, which includes sperm count, sperm motility, and sperm morphology. On the whole, up to 30% of infertile couples have unexplained infertility: infertility for which the underlying cause is unknown [6, 7]. The underlying cause of unexplained infertility may be oocyte- or sperm-related. Sperm-related causes of unexplained infertility are thought to include occult sperm abnormalities not detectable through routine semen analysis, but are often presumed after repeated failed cycles of intrauterine insemination (IUI) [8].

Sperm can go through several selection processes prior to traditional IVF, including density gradient centrifugation and swim-up protocols [9]. These processes can increase the concentration of sperm capable of fertilization, making it more likely to have success in traditional IVF when compared to IUI and natural conception. Nonetheless, some couples with male factor infertility have a decreased ability to generate embryos and pregnancies [10]. The men in these couples likely have reduced sperm function related to the failure of sperm capacitation. Therefore, at the intersection between infertility with a male factor and unexplained infertility lies a potential sperm-related cause of infertility—failure of sperm capacitation.

Sperm capacitation consists of the functional maturation of sperm membranes triggered by stimuli in the female genital tract. Capacitation results in a change in the pattern of sperm motility, known as hyperactivation. Capacitation precedes and is a precondition for the acrosome reaction and is required for fertilization. The timing of capacitation occurs differentially among men but consistently within men [11]. Because of this, capacitation timing can be utilized to personalize natural conception as well as assisted reproductive technologies (ART) by optimizing the timing for IUI relative to ovulation and timing of co-incubation in sperm and oocytes for in vitro fertilization (IVF).

The Cap-Score™ is an in vitro, laboratory-developed test designed to assess sperm capacitation. The Cap-Score™ evaluates the localization patterns of the ganglioside GM1 to assess the fertilizing ability of sperm. Sperm are incubated in a non-capacitating (non-Cap) medium and a medium containing capacitating stimuli (Cap). The sperm that respond to the capacitation stimuli are identified by specific GM1 localization patterns within their plasma membrane. The final readout—the “Cap-Score”—is the proportion of sperm within an ejaculate displaying the GM1 localization patterns reflecting capacitation [12].

There is little agreement on cutoffs for lower limits of semen variables in fertile men, and proposals have included the 10th centile [13–16], the 5th centile [17–19], and the 2.5th centile [20]. It has been demonstrated that Cap-Score™ results follow a normal distribution with 68% of values within one standard deviation (SD) of the mean, resulting in approximately 16% of observations being below one SD of the mean [21]. For the purposes of this study, a low Cap-Score™ was defined as a score of 27.6% which is one SD below the mean Cap-Score™ of a fertile population (*Z* score of − 1), a threshold that can be used to identify samples from men with impaired capacitation ability. We chose this to be conservative in order to minimize the risk that an individual with a borderline score would be identified as potentially having impaired sperm function. Such a cutoff has been reported in another population of men identified with sperm functional abnormalities as to expect them to be infertile in the general population [5] and has been used by others [22].

Details on the performance of the Cap-Score™ have been published elsewhere [12]. Briefly, sperm are collected, liquefied, and removed from the seminal plasma. The sperm concentration is adjusted to 10 × 10^6^/mL in total volume of 300 μL, and the samples are incubated in the presence or absence of “Cap” or capacitation stimulus. Following incubation, the cells are fixed and labeled with Alexa Fluor 488-conjugated cholera toxin beta subunit to visualize GM1 localization patterns within the sperm plasma membrane. The G_M1_ localization patterns are determined for 150 sperm and the Cap-Score is calculated as the proportion of sperm within the ejaculate having capacitated patterns.

The objective of this study was to assess the potential clinical and cost impact of the Cap-Score™. We estimated the differences in clinical pregnancy rates, live birth rates, and medical costs comparing the use of the Cap-Score™ with timed IUI (CS-TI) and the current standard of care (SOC) in which the Cap-Score™ is absent.

## Methods

We used decision-analytic modeling methods to estimate the cost and outcome implications of introducing the Cap-Score™ into the practice setting for unexplained infertility. The target population in the model is couples with unexplained infertility.

**Fig. 1:**
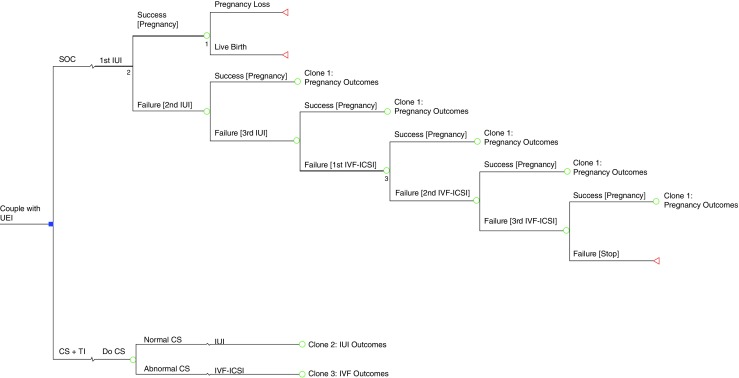
Decision tree of the outcomes of clinical management of unexplained infertility. UEI unexplained infertility, SOC standard of care, CS + TI cap-score + timed insemination, CS cap-score, IUI intrauterine insemination, IVF in vitro fertilization, ICSI intracytoplasmic sperm injection

We compared two possible clinical courses of action (comparators) (Fig. 1): (1) the Cap-Score and timed intrauterine insemination (CS-TI) and (2) the standard of care (SOC). In the SOC, we assumed that the couple would undergo three rounds of IUI followed by three rounds of IVF with intracytoplasmic sperm injection (ICSI). This is considered to be the general standard of practice when couples are paying and this threshold has been used by others in the literature [21]. In the CS-TI arm, the male would start by providing sperm for a Cap-Score™. A low Cap-Score™ was assumed to trigger three rounds of IVF with ICSI without IUI. A normal Cap-Score™ was assumed to trigger three rounds of timed IUI followed by three rounds of IVF with ICSI. The outcomes of the model were cumulative probability of live birth, cumulative probability of clinical pregnancy, total medical costs, IUI costs, and IVF costs.

### Probabilities

To estimate the probabilities of fertility-related events, we used data from publicly-available and published sources (Table 1). The probabilities of clinical pregnancy per cycle were modeled as maternal age-dependent and were obtained from the study by Stone et al. [23] for IUI and from the online database of the Society for Artificial Reproductive Technology (SART) [24] for IVF. The probability of men having a low Cap-Score™ was obtained from clinical studies [21]. The probability of clinical pregnancy with IUI following a normal Cap-Score™ was assumed to be independent of maternal age [25]. The probabilities of live birth by comparator, conditional upon being clinically pregnant, were also modeled as maternal age-dependent, and were obtained from the study by Stone et al. [23] and the SART database [24] for IUI and IVF, respectively. For sensitivity analysis ranges, we used 95% confidence intervals when available, and when unavailable, we used ranges equivalent to +/− 20% for the Cap-Score, and +/− 50% for variations in the probability of IUI success.

**Table 1:** Parameter estimates used in the model

Parameter	Baseline	Sensitivity range	Reference^a^
Costs			
IUI	$2550	$1275–$3825	Expert opinion
IVF-ICSI	$17,651	$8825–$26,476	[28]
Probabilities			
Low Cap-Score Test	0.336	0.269–0.403	[35]
Clinical pregnancy (per cycle)			
With IUI in SOC			
< 35 years	0.161	0.081–0.242	[23]
35–37 years	0.136	0.068–0.204	[23]
38–40 years	0.118	0.059–0.177	[23]
41–42 years	0.068	0.034–0.102	[23]
> 42 years	0.035	0.018–0.053	[23]
With IUI after Cap-Score Test	0.400	0.320–0.480	Expert opinion
With IVF-ICSI			
< 35 years	0.506	0.405–0.607	[24]^b^
35–37 years	0.445	0.356–0.534	[24]^b^
38–40 years	0.320	0.256–0.384	[24]^b^
41–42 years	0.224	0.179–0.269	[24]^b^
> 42 years	0.121	0.097–0.145	[24]^b^
Live birth (per cycle)^c^			
With IUI			
< 35 years	0.559	0.279–0.838	[23]
35–37 years	0.495	0.248–0.743	[23]
38–40 years	0.462	0.231–0.692	[23]
41–42 years	0.400	0.200–0.600	[23]
> 42 years	0.386	0.193–0.579	[23]
With IVF-ICSI			
< 35 years	0.879	0.854–0.903	[24]^b^
35–37 years	0.829	0.793–0.865	[24]^b^
38–40 years	0.772	0.722–0.822	[24]^b^
41–42 years	0.594	0.500–0.688	[24]^b^
> 42 years	0.496	0.324–0.670	[24]^b^

^a^Represents 95% confidence interval or low and high estimates from multiple studies where data are available and +/− 50% for both costs and probabilities where data are unavailable or uncertainty is substantial

^b^Primary outcome per intended egg retrieval with all cycles as the denominator

^c^Conditional upon clinical pregnancy

### Costs

The perspective of the cost analysis was that of the social opportunity cost: i.e., all the costs of ART are included as reflected in market transaction prices whether paid by patients or third-party payers. This perspective was used because approximately 20% of US states mandate full or partial payments for ART, and most patients must pay a proportion or all of the costs of ART out of pocket [26]. To estimate the costs of IUI, given the wide state and regional variation, we used multiple sources including a public website [27] and expert opinion. The baseline cost of IUI considers the average cost of Clomid medication, injectable fertility medications for injectable follicle stimulating hormone (FSH) cycles, and costs of monitored injectable FSH cycles (blood tests and ultrasounds). The cost of IVF was obtained from the study by Chambers et al. [28] adjusted to 2016 US dollars. This cost includes the cost of ICSI. The Cap-Score Test™ has not yet been launched, and no price has been established. Therefore, we are only able to report cost-offsets that would accrue as savings gains, whatever the ultimate price. All costs are in 2016 US dollars and are shown in Table 1.

### Analyses and sensitivity analyses

The base case analysis consisted of estimating the mean cumulative probability of pregnancy, the mean cumulative probability of live birth, IUI costs, IVF costs, and total costs comparing CS-TI and SOC. As a measure of the precision of estimates, we calculated 95% credibility intervals (CIs) by defining probability distributions for each parameter estimate and using 10,000 runs of Monte Carlo simulation. Monte Carlo simulation is a method of analysis of parameter uncertainty in which multiple runs of a model are estimated, with each run drawing from the distribution of each of a set of parameter values. The results of the multiple runs produce a distribution of outcome values, thereby allowing analysts to produce estimates of the impact of parameter uncertainty on model results. We used beta distributions for probabilities and normal distributions for costs. We also assessed parameter uncertainty by performing univariate sensitivity analyses, varying individual parameters with all other parameters in the model held constant.

## Results

### Baseline analysis

The results of the baseline analysis are presented in Tables 2 and 3. Compared to SOC, CS-TI is projected to increase the cumulative clinical pregnancy rates across all age groups. The magnitude of this increase ranges from a 1% increase in couples with women under 35 years to 26% in couples with women over 42 years. Compared to SOC, CS-TI led to marginal reductions in the cumulative live birth rate ranging from 1% in couples with women of between 38 and 40 years and 3% in couples with women under 35 years of age. In couples with women over 40 years of age, CS-TI results in increased cumulative live birth rates of between 3% in couples with women of 41 and 42 years of age and 7% in couple with women over 42 years of age.

**Table 2:** Mean (95% CI) cumulative live birth rate and clinical pregnancy rate comparing SOC to CS-TI

Age (years)	Clinical pregnancy rate			Live birth rate		
	SOC	CS-TI	Δ	SOC	CS-TI	Δ
< 35	92.88% (86.97–96.65%)	94.22% (89.59–97.12%)	1.34%	68.54% (54.11–79.72%)	66.16% (51.09–80.30%)	− 2.38%
35–37	88.97% (81.94–93.81%)	91.80% (87.08–95.48%)	2.83%	61.90% (50.74–71.89%)	58.72% (45.15–71.73%)	− 3.18%
38–40	78.43% (70.32–85.26%)	84.93% (79.51–89.55%)	5.29%	50.81% (41.65–59.77%)	49.42% (36.27–62.74%)	− 1.13%
41–42	62.17% (54.16–69.38%)	77.60% (64.76–84.65%)	15.43%	33.23% (26.45–40.26%)	35.99% (26.76–47.05%)	2.76%
> 42	38.97% (33.03–45.10%)	64.83% (55.44–71.04%)	25.86%	17.71% (12.37–24.61%)	24.44% (17.11–39.53%)	6.73%

*CI* credibility interval, *SOC* standard of care, *CS-TI* Cap-Score™ with timed intrauterine insemination, *Δ* change

**Table 3:** Mean (95% CI) IUI costs, IVF costs, and total costs comparing SOC to CS-TI

Age (years)	IUI costs			IVF costs			Total costs		
	SOC	CS-TI	Δ	SOC	CS-TI	Δ	SOC	CS-TI	Δ
< 35	$6484 ($3217–$9820)	$5889 ($2932–$8735)	− $595	$18,118 ($8385–$29,724)	$14,708 ($6929–$23,111)	− $3410	$24,603 ($14,039–37,358)	$20,597 ($12,345–$30,331)	− $4006
35–37	$6657 ($3337–$10,095)	$5889 ($2773–$8599)	− $768	$21,210 ($10,087–$34,391)	$15,766 ($7528–$24,214)	− $5444	$27,866 ($15,887–$41,803)	$21,655 ($12,839–31,034)	− $6211
38–40	$6783 ($3318–$10,272)	$5889 ($3041–$8929)	− $894	$25,946 ($12,477–$40,924)	$18,129 ($9208–$27,870)	−$6364	$32,729 ($18,657–$48,205%)	$24,019 ($14,340–$35,285)	− $7128
41–42	$7142 ($3600–$10,786)	$5889 ($3567–$9657)	− $1253	$33,983 ($16,705–$52,003)	$20,124 ($9567–$31,450)	− $13,859	$41,125 ($23,697–$59,210)	$26,014 ($15,597–$34,200)	− $15,111
> 42	$7385 ($3678–$11,100)	$6019 ($3221–$9444)	− $1366	$42,060 ($21,423–$63,241)	$24,237 ($11,756–$36,600)	− $17,823	$49,445 ($28,240–$71,335)	$30,256 ($18,622–$43,253)	− $19,189

*CI* credibility interval, *SOC* standard of care, *CS-TI* Cap-Score™ with timed intrauterine insemination, *Δ* change

Compared to SOC, CS-TI is projected to reduce cumulative mean number of IUIs and IVFs as well as the total costs of fertility treatment across all age groups. Mean IUI cost savings are projected to vary from $595 in couples with women under 35 years of age to $1366 in couples with women over 42 years of age. Mean IVF cost savings are projected to vary from $3410 in couples with women under 35 years of age to $17,823 in couples with women over 42 years of age. Mean total cost savings are projected to vary from $4000 in couples with women under 35 years of age to $19,100 in couples with women over 42 years of age.

### Sensitivity analysis

The projected change in the cumulative probability of clinical pregnancy comparing CS-TI and SOC was most sensitive to the probability of clinical pregnancy with IUI in the absence of the Cap-Score™ for couples with women under 42 years of age and most sensitive to the probability of a low Cap-Score™ for couples with women over 42 years of age.

The projected change in the cumulative probability of live births comparing CS-TI and SOC was most sensitive to the probability of clinical pregnancy with IUI in the absence of the Cap-Score™ for couples with women under 35 years of age and most sensitive to the probability of a live birth with IUI for couples with women over 35 years of age.

The projected reduction in IUI costs comparing CS-TI and SOC was most sensitive to the probability of clinical pregnancy with IUI in the absence of the Cap-Score™ for couples with women under 40 years of age and most sensitive to the cost of IUI for couples with women over 40 years of age.

The projected reduction in IVF costs comparing CS-TI and SOC was most sensitive to the probability of clinical pregnancy with IUI in the absence of the Cap-Score™ for couples with women under 40 years of age and most sensitive to the cost of IVF for couples with women over 40 years of age.

The projected reduction in total costs comparing CS-TI and SOC was most sensitive to the probability of clinical pregnancy with IUI in the absence of the Cap-Score™ for couples with women under 40 years of age and most sensitive to the cost of IVF for couples with women over 40 years of age.

Thus, 55% of the 20 different outcomes estimated, including age-group-specific outcomes, were most sensitive to the probability of clinical pregnancy with IUI in the absence of the Cap-Score™.

## Discussion

In this study of the potential clinical and cost impact of introducing the Cap-Score™ for clinical management of unexplained infertility, we found an overall increase in clinical pregnancy rates, an increase in live birth rates in couples with women over 40 years of age, and across the board cost savings comparing the Cap-Score™ and timed insemination with the current standard of care in which the Cap-Score™ is absent. The increase in clinical pregnancy rates, live birth rates, and cost savings are higher in older age categories, suggesting increasing clinical and economic value of the Cap-Score™ with increasing age.

The results were generally robust to sensitivity analyses, i.e., changing parameters through their plausible ranges did not result in substantial changes to results. The majority of outcomes were most sensitive to the probability of clinical pregnancy with IUI in the absence of the Cap-Score™. This variable is not directly related to the Cap-Score™. The expected cumulative probability of live birth in women over 35 years was most sensitive to the live birth rate with IUI, reflecting the importance of maintenance of successful pregnancies in older women. As expected, the expected difference in the cost of IUI between comparators was most sensitive to the cost of IUI, and the expected difference in the cost of IVF and expected difference in total cost between comparators were both most sensitive to the cost of IVF.

The potential increase in clinical pregnancy rates, live birth rates, and cost savings attributable to the Cap-Score™ consistently increase with age because of decreasing probability of clinical pregnancy with age when women undergo IUI in the absence of a Cap-Score and the constant probability of clinical pregnancy with age when women undergo IUI following a Cap-Score. The Cap-Score™ therefore increases in clinical and economic value for couples who would otherwise have a hard time conceiving. The model predicts a potential modest decrease in live birth rates for women under 40 years of age. This is due to the fact that population-level rates of IUI success are projected to be substantially lower than the rates of IUI success in women receiving the Cap-Score™ algorithm in clinical practice. Women receiving IUI under a Cap-Score™ algorithm would have a higher chance of IUI success because a proportion of women whose partners have abnormal Cap-Scores™ (and therefore have a lower chance of IUI success) would go directly to IVF-ICSI, leaving patients with a higher chance of IUI success.

The Cap-Score™ has the potential to change the management of male factor infertility and infertility of unknown origin by increasing access, improving outcomes, and saving money. Given the substantial unmet need for infertility treatment in the USA—over 50% of the need for fertility treatment is unmet [29, 30], and just over 60% of nulliparous women in the USA with current fertility problems have ever used infertility services [4]—the Cap-Score™ is a potential avenue for increased access. Increased access to ART will likely result due to cost savings. Because most patients who undergo fertility treatment, in general, and ART, in particular, have to pay out of pocket due to lack of insurance or under insurance [26, 31], cost savings as a result of the Cap-Score™ may lead to a larger number of infertile couples being able to afford and therefore seeking care for infertility. Additionally, because treatment for infertility exerts a substantial burden due to psychological stress, lost work, and travel costs and time [31], improved outcomes attributable to the Cap-Score™ may encourage patients to seek care, thereby increasing access.

The ultimate value of the Cap-Score™ will be depend on the price of the test as a key factor in its cost-effectiveness. Currently, the test has not yet been launched, and no price has been established. Once the price is established, a comparison of CS-TI to SOC will lead to either a cost-saving or cost-increasing scenario accompanied by increased benefit, and an economic cost-effectiveness assessment of value will require the use of a benchmark or threshold willingness to pay for additional outcome. Because couples seek fertility treatment to have children, the most important outcome is live births. To be able to make decisions about the value of the Cap-Score™ will require estimates of individual’s willingness to pay for a live birth. A previous contingent valuation study based on hypothetical scenarios has estimated an ex-ante and ex-post statistical willingness to pay for a baby at $1.8 million and $178,000, respectively [32], suggesting that the Cap-Score will add substantial value. Another study demonstrated that the couples were willing to pay more for a child than the calculated direct medical costs [33]. Another study using contingent valuation has also demonstrated a high willingness to pay for ovarian stimulating hormone in the treatment of infertility [34].

One limitation of this study is that since the Cap-Score™ is a novel technology, there are limited data on its use in the clinical setting. This limits the evidence available to support the assumptions about the value of two key parameters—probability of a low Cap-Score™ (compared to a normal Cap-Score™) and the probability of clinical pregnancy with IUI following a normal Cap-Score™. This model could be refined without changing the basic structure and the estimates updated once there is diffusion of the Cap-Score™ into clinical practice and better data are available.

## Conclusion

In this analysis, we project the potential clinical and cost impact of the Cap-Score™ as a clinical tool for management of unexplained infertility. The Cap-Score™ is projected to improve pregnancy and birth outcomes and save money—thereby potentially increasing access to management of infertility.
